# Current News and Opinion

**Published:** 1887-06

**Authors:** 


### Current News and Opinion.

### THE WAITE TESTIMONIAL FUND.

*Dr. W. C. Barrett:*

Dear Sir—Please find enclosed receipt for £81 17s. 8d. on account of the Waite fund, a noble contribution from our American confrères.

I cannot find language to describe to you the high appreciation and warm sentiments that were expressed on all hands when the amount was announced at our meeting.

This truly spontaneous sympathy with us in so worthy a cause will do more to bind the profession together in a loving rivalry for good than anything that has gone before.

Thanking you sincerely for the hearty way in which the matter was taken up,

Believe me, dear sir, yours faithfully,

Thos. Murphy.
Hon. Treas.

Springfield, St. George's Road,
Bolton, May, 9, 1887.

---

10 Oxford Street, Liverpool, May 3, 1887.

*Dr. W. C. Barrett:*

Dear Sir—Kindly permit me a brief space in your journal to record my heartfelt appreciation of the brotherly love displayed by my American friends. The warm-hearted hospitality and generous consideration of American brethren won my heart twenty-three years ago, when, a perfect stranger, I went to study at the Philadelphia Dental College. From that time to the present, one of the strongest desires of my life has been to cultivate and promote a sentiment of true brotherhood between England and America. The most gratifying aspect of your practical and liberal sympathy is the evidence it affords of a deep, earnest and growing recognition of the indissoluble ties which bind together these two mighty nations.

As a personal gift, I have no claim whatever to the very handsome contribution made by America toward the splendid testimonial presented by my English brethren. But as a declaration of international courtesy, as a tribute of professional good-will, and chiefly as an expression of fraternal affection and sympathy, I rejoice to accept and to acknowledge the offering, prompted by the purest and the holiest feelings of which our common nature is capable.

Whatever the shortcomings of our profession in other respects, it must at least be conceded that we have risen to the honor and delight of gracefully endeavoring to comfort and encourage one another in time of trial or difficulty. Professional spirit may extend and amplify this principle, but it cannot be excelled, and no other profession, so far as I am aware, has ever furnished a brighter example of true Christian emotion than that afforded by this prompt and hearty participation of brethren on both sides the Atlantic.

I am deeply sensible, dear Dr. Barrett, of my great weight of obligation for the vigorous manner in which you grasped the opportunity, and not less for the considerate and delicate tenderness with which you evoked this unparalleled expression of brotherly kindness. I assure you, your English brethren are deeply moved by this exhibition of unity, fraternity and sympathy. You have set an example they will not be slow to follow. Such principles over-ride all barriers, survive all changes, and endure even to eternity. Though disabled, I am not disheartened, and I trust still to have some opportunity of bearing witness in behalf of that which may subserve the best interests of the profession we all love.

Believe me, dear sir, with every assurance of esteem and gratitude, to remain, Yours very truly,

W. H. WAITE.

---

### CONNECTICUT VALLEY DENTAL SOCIETY.

The Connecticut Valley Dental Society adopts quite an innovation this year by going to Montreal, Canada, to hold its semi-annual meeting, which opens at the Windsor Hotel, July 19th, and continues four days. The feast of good things offered to the profession will include papers from Prof. G. V. Black, Jacksonville, Ill., Prof. Chas. Mayr, of Springfield, and Prof. R. R. Andrews, of Cambridge, the latter's paper to be illustrated with the stereopticon.

The dentists of Montreal already evince much enthusiasm in regard to the meeting, and will do all in their power to make it a success. The programme will be so arranged as to combine business with pleasure, and opportunities will be given to visit the various points of interest in and about Montreal, including a sail down the wonderful Lachine Rapids, while excursions at reduced rates will be arranged to Quebec, Montmorency Falls, and to the Saguenay; also to Ottawa. Rates at the Windsor will be reduced to $3.00 and $3.50 per day to those attending the meeting. Round trip tickets from Springfield, good for two weeks, will be $12.00. Rates from other points cannot be announced until July 1st. A cordial invitation is extended the profession to attend, and *a special request is made that each one bring his wife with him.* If those who intend going will correspond with the secretary, they will save themselves much trouble in regard to securing rooms, etc.

GEO. A. MAXFIELD, D. D. S., Secretary,
Holyoke, Mass.

---

### FIFTH DISTRICT DENTAL SOCIETY.

The nineteenth annual meeting of the Fifth District Dental Society of the State of New York was held at Utica, N. Y., April 12th and 13th, 1887. Three new members were received, and the following officers elected for the ensuing year:

*President*—C. H. Bennett, Waterville.
*Vice-President*—B. T. Mason, Phœnix.
*Recording Secretary*—C. J. Peters, Syracuse.
*Correspondent*—G. H. Butler, Skaneateles.
*Treasurer*—A. R. Cooke, Syracuse.
*Librarian*—A. Retter, Utica.

C. J. PETERS, Sec'y Fifth D. D. S.

### DENTAL JOURNALS WANTED.

The editor of this journal will pay cash for the following numbers of dental journals, or an exchange will be made with those who desire to complete their own files.

THE DENTAL REGISTER.

Vol. III, Nos. 1, 2, 3.

Vol. VI, Nos. 1, 2, 3, 4.

AMERICAN JOURNAL OF DENTAL SCIENCE.
(Third Series.)

Vol. VII, Nos. 7, 10.
Vol. VIII, Nos. 6, 7.

---

THE LONG PENDING SUIT between Drs. John Evans and Thomas W. Evans, the two famous dentists of Paris, relative to the bearing of the name of Evans by the former, has been definitely decided by the courts in favor of Dr. John Evans, and his adversary has been condemned to pay all the costs. Dr. John Evans had taken the name of D'Oyley for use in private life, so his uncle contested his right to continue to call himself Evans in his business relations. "Medical and dental journals please copy."—*Galignani's Messenger.*

Certainly, we will "copy!" But it would appear by this extract from the Paris paper that Dr. John is ashamed of his vocation, and in private life desires to be dissociated from the professionally famous name of Evans. Whatever may be the possible faults of Dr. T. W. Evans, it was never charged against him that he was guilty of any such act of poltroonery and snobbishness, and it seems a decided pity that his suit to restrain his unworthy relative from using the name for sordid purposes alone, has not been successful.

---

A FLUX that is exceedingly useful in bridge work is prepared as follows: Put in a cup—

| | |
|---|---|
| Boracic Acid, | 1 oz. |
| Ammonia, | ½ oz. |
| Carbonate of Ammonia, | ½ dwt. |
| Bicarbonate of Soda, | 2 dwt. |
| Water, | 4 oz. |

Boil until the fumes of ammonia are no longer given off.

Coat the bridge or other work all over the gold with the flux. Heat it over a spirit lamp to dry it on. Give it another coat, if needed, leaving no part exposed. Then scrape off where it is desired that the solder shall flow, and it will go nowhere else. The work will come out of the heating as bright as when it went in, and the solder will be smooth. The polished surfaces will not be corroded or blackened.

H. W. HOWE, D. D. S.

---

THE THIRD INSTALLMENT of the "Unpublished Letters of Thackeray," which will appear in *Scribner's Magazine* for June, will contain a four-page letter in fac-simile, with a pen-and-ink sketch of Jules Janin by Thackeray. A number of other Thackeray drawings will be reproduced from the rare collection privately printed for Sir Arthur Elton. The letters are said to be full of that humor, pathos, and kindly sentiment which have made Thackeray so lovable as writer and man.

MR. SMALE, of England, relates the case of a boy aged eight, who had the right upper central incisor twisted so that the mesial surface presented toward the lip. The tooth was grasped firmly by a pair of straight-bladed forceps and twisted into a good position, care being used to press the tooth firmly into the socket during the operation. It was tied to the surrounding teeth with silk twist, to prevent its returning to the old position. A week afterwards it was quite firm, the tooth could be tapped, and the boy could distinguish between hot and cold applications. There was no discoloration, and the gum was healthy. Torsion, says Mr. Smale, may be used freely before the patient arrives at the age of twelve years, and should always be done at one operation. It is only applicable to incisors.

---

IN THE CASE of Dr. Frederick B. Mandeville, of Newark, N. J , who, in compliance with a contract, applied for an injunction to restrain his former assistant, Dr. George W. Harmon, from practicing medicine in Newark, the Vice-Chancellor has refused to grant the injunction. The Vice-Chancellor says that the covenant by which Dr. Harmon bound himself not to open an office in Newark after quitting the service of Dr. Mandeville, was unreasonable and therefore void. Professional skill, experience and reputation, are things that cannot be bought or sold. They constitute part of the individuality of the particular person, and die with him.

That may be New Jersey law, but it is neither reason nor justice.—EDITOR.

---

DR. L. W. BRISTOL, of Lockport, has read before the Seventh and Eighth District Dental Societies historical papers and recollections of his long professional life, which were published in the INDEPENDENT PRACTITIONER and have been very extensively quoted by our contemporaries. Whether by error or of malice aforethought, they have usually been credited to the journal which first cribbed them from us. It is a little matter, but it is becoming so common that it is time for us to enter a protest, and perhaps call upon some one for an explanation, for the habit of quoting from this jourual and giving credit to another seems to be growing on our brother editors.

---

A DECREE has been officially advertised in Germany, in the *Deutsche Medizinal-Zeitung*, ordering all persons not possessed of an approved medical qualification to discontinue within three months the use of the name "American Dentist" on signs, cards, etc. The reason given is that it may produce the impression that the bearer is an approved practitioner of medicine, but there is no doubt that it is intended as a discrimination against American dentists, and is another exhibition of the dislike of the German government to Americans, and the jealousy of American dentists. Our dental degree is thrown out because of the irregularity too often connected with its bestowal.

---

"SPARKLING AND BRIGHT," better than champagne. The Underwood Spring water is highly charged with carbonic acid gas and is a most refreshing drink for warm weather, which the weary dentist will know how to appreciate when he has tested its qualities.

PYTHAGORAS asserted that the seat or location of the soul is the encephalon; Eristratus, the meninges; Herophilus, the great ventricle; Servetus, the aqueduct of Sylvius; Arontius, the third ventricle; Des Cartes, the pineal gland; Sœmmering, the liquid contained in the encephalon; Aristotle, the heart; others the origin of the spinal cord, the corpus callosum, the corpore striata, etc. Empedocles believed that it circulated through the blood.

---

DR. HERBST, instead of using a clamp for holding the rubber dam in position, cuts the point from a common pin, flattens it a little with a hammer, curves it to make it partially conform to the shape of a tooth, and inserts it next the tooth to be filled. The rubber dam is then hitched over it, and thus held until the ligatures can be adjusted. He thus reduces the discomfort to the minimum point.

---

DR. JAMES G. PALMER, formerly of New Brunswick, N. J., has removed to New York City, and may be found at No. 112 West 38th Street. Dr. Palmer has always been active in society and professional work, and his removal to the metropolis will give him a broader field. "'Tis not in mortals to command success," but Dr. Palmer will certainly deserve it.

---

ALMOST EVERYONE can enjoy a good joke, even if it is at some one's else expense, and *The Western Dental Journal* makes an especially good point on *Archives of Dentistry*, apropos to the Fifty editors and Forty-three sub-editors or correspondents of the latter, when it intimates that *Archives* is publishing its subscription list upon the title page.

---

AN ECLECTIC MEDICAL COLLEGE, managed by Dr. Samuel York, of Lewiston Me., has, it is charged, engaged in the selling of diplomas to students who had been engaged in study for less than a day. York's diplomas, besides many other things, covered "operative dentistry," but the students did not have the opportunity even to see a tooth extracted.

---

"THE ARLINGTON" has been selected as the headquarters of the Section of Dental and Oral Surgery in the International Medical Congress, which opens September 5th at Washington. The terms to delegates will be three and four dollars per day for each person, the price depending upon location. Rooms should be secured early.

---

IT IS ESTIMATED that in 1881 medical books formed one-thirtieth of the whole mass of the world's literature. The number of separate volumes was more than 120,000, and of pamphlets a quarter of a million. Since that time medical literature has increased at the rate of 1,500 volumes and 2,500 pamphlets annually.

---

DR. D. G. BRINTON has retired from the editorship of the *Medical and Surgical Reporter*. But such an accomplished and experienced writer cannot keep out of the editorial harness, and he gives notice that he will shortly establish a new medical journal. It would be a great professional loss should Dr. Brinton retire altogether.

MONTREAL has received the munificent gift of $1,000,000 from two wealthy men who have been knighted by the Queen, and who propose to endow a hospital to be called "The Royal Victoria Hospital." What a pity that the Queen cannot knight some of our wealthy men, if it would produce like results.

THE POSTAL LAW makes it larceny if a man takes a newspaper or periodical from the postoffice and then refuses to pay for it. It does not matter whether he shall have subscribed for it or not. If he receives and accepts it he must pay, or be liable to a criminal prosecution.

THE LEGISLATURE of Alabama has passed a stringent dental law which has been approved by the Governor. The Arkansas Dental Association was organized in January last, and the first work it set itself about was to organize the profession through an appropriate law.

DR. GEO. H. CUSHING, of Chicago, has removed from No. 34 Monroe Street to No. 96 State Street.

Dr. T. W. Brophy has removed from No. 125 State Street to the same number. Dr. W. W. Allport has removed to the Argyle Building.

DR. JAMES W. WHITE, editor of *The Cosmos*, has been appointed by the Mayor of Philadelphia, President of the Board of Charities and Correction. That Dr. White will worthily fill the office, no one who knows him will doubt.

THE NEW YORK DENTAL M'F'G CO., as may be seen by its advertisement, has been absorbed by the S. S. White Dental M'f'g Co., and will be known to dentists no more. The latter company "gathers them in" gradually, but surely.

THE AMERICAN MEDICAL ASSOCIATION will hold its thirty-eighth annual meeting in Chicago, Ill., commencing Tuesday, June 7th, and continuing four days. Reduced rates for members have been secured on most of the railways.

THE MISSOURI DENTAL SOCIETY meets at Kansas City, June 21st to 24th. The energetic President, Dr. Conrad, is moving all the Mississippi Valley to secure a successful meeting.

DR. J. H. MARTINDALE, of Minneapolis, Minn., sails early in June for Europe, for a tour of two or three months. His time will mostly be spent in France and Germany.

THE GERMAN ZAHNARZTLISCHES INSTITUTE, Berlin, has proved a great success, the students of the last semestre numbering 139 matriculants.

THE OJCZYZNA is a semi-weekly paper published in Buffalo, of which we have received a copy marked "Please X." Declined, with thanks.

AN EXCELLENT PICKLE for gold work may be prepared from the following formula: Oxalic acid, ½ oz.; sulphuric acid, 1 oz.; water, 6 oz.

DR. T. B. WHEELER, formerly of Chicago, has gone to Europe to enter upon practice. He intends to locate in Paris.

JOHNS HOPKINS University has an endowment of about $5,000,000. Last year it yielded a net income of $226,000.

